# Combining In Vitro, In Vivo, and In Silico Approaches to Explore the Effect of *Ceratonia siliqua* and *Ocimum basilicum* Rich Phenolic Formula on Lipid Metabolism and Plasma Lipoprotein Oxidation in Mice Fed a High-Fat Diet: A Follow-Up Study

**DOI:** 10.3390/metabo15010036

**Published:** 2025-01-10

**Authors:** Mohammadine Moumou, Amani Tayebi, Abderrahmane Hadini, Omar M. Noman, Abdulsalam Alhalmi, Hamza Ahmoda, Souliman Amrani, Hicham Harnafi

**Affiliations:** 1 Laboratory of Bioresources, Biotechnologies, Ethnopharmacology and Health, Faculty of Sciences, University Mohammed First, Oujda 60000, Morocco; mohammadine.moumou@ump.ac.ma (M.M.); amani.tayebi@ump.ac.ma (A.T.); abderrahmane.hadini@ump.ac.ma (A.H.); samrani@ump.ac.ma (S.A.); 2 Department of Pharmacognosy, College of Pharmacy, King Saud University, P.O. Box 2457, Riyadh 11451, Saudi Arabia; onoman@ksu.edu.sa; 3 Department of Pharmaceutics, School of Pharmaceutical Education and Research, Jamia Hamdard, New Delhi 110062, India; asalamahmed5@gmail.com; 4 Universitätsklinik für Viszerale Chirurgie und Medizin, Universität Bern, 3010 Bern, Switzerland; h.ahmoda@bluewin.ch

**Keywords:** *Ceratonia siliqua*, *Ocimum basilicum*, HPLC-DAD, polyphenols, lipid metabolism

## Abstract

Background/Objectives: Hyperlipidemia is a serious risk factor for cardiovascular diseases and liver steatosis. In this work, we explored the effect of an herbal formula (CBF) containing immature *Ceratonia siliqua* pods and *Ocimum basilicum* extracts on lipid metabolism disorders and lipoprotein-rich plasma (LRP) oxidation in mice. Methods: The phenolic composition was determined using HPLC-DAD analysis. The antioxidant activity was studied using various in vitro methods. Acute toxicity was evaluated in mice. Importantly, the effect of the CBF on lipid metabolism disorders was investigated in a high-fat diet (HFD) hyperlipidemia mouse model. An in silico study was carried out to predict underlying mechanisms. Results: The HPLC analysis revealed gallic acid, cinnamic acid, and naringenin as major phenolics of the carob pod aqueous extract. Concerning the basil hydro-ethanolic extract, rosmarinic, chicoric, caftaric, and caffeic acids were the main phenolics. Accordingly, the CBF prevented LRP oxidation in a concentration-dependent manner. This formula is not toxic in mice (LD_50_ > 2000 mg/kg body weight). Moreover, animals administered the CBF at 200 mg/kg/day presented a significant decline in their body weight gain, adipose tissue weight, plasma total cholesterol, low-density lipoprotein cholesterol (LDL-C) level, and glycaemia after 10 weeks’ treatment. Accordingly, the CBF decreased the plasma atherogenic index and the LDL-C to HDL-C ratio and reduced the level of fats accumulated in the liver. The molecular docking study revealed that chicoric, rosmarinic, and caftaric acids, and naringenin bound particularly strongly to many proteins involved in the regulation of lipid and cholesterol metabolism. This includes the HMG-CoA reductase, PPARα/γ, PCSK9, Cyp7a1, and ATP-citrate lyase. Conclusions: The CBF could be a good source of natural supplements, functional foods, and pharmaceuticals effective in managing hyperlipidemia and oxidative stress and preventing their related cardiovascular disorders.

## 1. Introduction

Hyperlipidemia is a serious metabolic dysfunction characterized by high blood levels of total cholesterol (TC), LDL-C, triglycerides (TG), and/or low blood concentration of high-density lipoprotein cholesterol (HDL-C) [1,2]. This metabolic disorder is the leading cause of cardiovascular diseases (CVD), including atherosclerosis and coronary heart diseases [3,4]. Indeed, there is a strong association between elevated LDL-C blood level and the incidence of CVD [5]. In contrast, many studies have shown that lowering the blood cholesterol level, especially the LDL-C one, plays a key role in maintaining cardiovascular health [6,7]. Additionally, overexpression of oxidative stress mediators, particularly reactive oxygen species (ROS), could induce significant damage to biomolecules. Oxidative stress caused the transformation of LDL (the major carrier of cholesterol in the blood) from its native form into an oxidized one [8]. This phenomenon plays a critical role in the initiation of atheroma plaque formation in medium- and large-caliber arteries, which is the early stage of atherosclerosis [7,9]. Indeed, the liver plays a crucial role in lipid metabolism. However, hyperlipidemia induces lipid droplet accumulation in hepatocytes, consequently leading to the development of non-alcoholic fatty liver diseases (NAFLD), such as hepatic steatosis and cirrhosis [10].

Although there are many drugs used to treat hyperlipidemia, like statins and fibrates, their various adverse or side effects are the major drawbacks of their utilization [11,12]. Currently, there is a growing interest among researchers in developing natural supplements to manage hypercholesterolemia and its related CVD [13]. Among the natural components, polyphenols constitute the most diverse family of phytochemicals with various health benefits [14]. These molecules are well known for their high capacity to neutralize free radicals and prevent oxidative stress-related diseases [15]. Incorporation of polyphenols in the diet has been linked to many health advantages, like preventing hyperlipidemia, obesity, diabetes, and related metabolic disorders [16]. In Morocco, *Ceratonia siliqua* and *Ocimum basilicum* are two medicinal plants used traditionally to manage hypercholesterolemia, diabetes, and other related metabolic diseases [17,18]. Previous phytochemical studies have shown that *C. siliqua* pods and *O. basilicum* aerial parts contain a variety of phytochemicals. Gallic acid, naringenin, quercetin, and catechin dominate the carob pods phenolic profile [19,20]. Moreover, unripe carob pods contain higher amounts of total polyphenols, flavonoids, and tannins than mature or ripe ones, which gives them much higher health benefits in terms of antioxidant and anti-inflammatory activities [19,21,22]. *O. basilicum,* commonly known as sweet basil, is a medicinal herb belonging to the *Lamiaceae* family. This plant has been used traditionally for culinary purposes and also to manage cardiovascular disorders, diabetes, and gastrointestinal problems [23]. Studies that have investigated the phenolic composition of *O. basilicum* from different regions of the world have shown that the plant’s aerial parts contain a variety of phenolic compounds. The most reported were rosmarinic, caftaric, chicoric, and caffeic acids [24,25,26]. Previous studies conducted by our team have demonstrated the efficacy of basil extracts in ameliorating hyperlipidemia and liver lipid accumulation in acute and chronic animal models [25,27,28,29]. Similarly, in our recently published work, we have demonstrated the considerable capacity of immature carob pod aqueous extract in preventing plasma lipoprotein oxidation in vitro and ameliorating hypercholesterolemia, hypertriglyceridemia, and liver lipid accumulation in mice [30].

In this study, the phenolic composition of *C. siliqua* unripe pod aqueous extract and *O. basilicum* hydro-ethanolic extract was analyzed using the HPLC-DAD method. Indeed, the antioxidant activity of a combination of the two plants was evaluated in vitro using the DPPH radical scavenging activity, the ferric reducing power, and the oxidation of LRP. Importantly, the hypolipidemic activity was studied in a high-fat diet (HFD)-fed mice model for 10 weeks using fenofibrate as a positive control. The effects of the combination of the two plants on plasma lipid profile and glycaemia, besides the atherogenic index and liver lipid accumulation, were investigated. Furthermore, the acute toxicity was evaluated in mice administered orally a single high dose of the CBF (2000 mg/kg).

Nowadays, molecular docking is considered a powerful tool in drug design and discovery. This approach helps researchers predict the binding patterns between a ligand (small molecule) and a target (receptor) [31]. In the current work, we used this method to predict the possible mechanisms involved in the hypolipidemic effect of the CBF main bioactive constituents. Thus, the interaction between the CBF phenolic constituents and the main proteins involved in controlling lipid and cholesterol metabolism (12 targets including HMG-CoA reductase, PPARα/γ, ATP-citrate lyase, Cyp7a1, and PCSK9) was studied using in silico molecular docking tools.

## 2. Materials and Methods

### 2.1. Chemicals and Drugs

Butylated hydroxyanisole (BHA), 2,2-diphenyl-1-picrylhydrazyl (DPPH), catechin, copper sulfate (CuSO_4_), tiobarbituric acid (TBA), rosmarinic acid, chicoric acid, quercetin, caftaric acid, vanillin, caffeic acid, syringic acid, rutin, gallic acid, naringenin, cinnamic acid, sodium carbonate (Na_2_CO_3_), ascorbic acid, potassium ferricyanide (K_3_Fe(CN)_6_), ferric chloride (FeCl_3_), trichloroacetic acid, aluminum chloride (AlCl_3_), and Folin–Ciocalteu reagent were purchased from MERCK (Lyon, France). All chemicals, including solvents, were of analytical grade.

### 2.2. Preparation of Extracts

#### 2.2.1. Preparation of *C. siliqua* Pod Aqueous Extract

In order to prepare the crude extract of *C. siliqua* pods, unripe pods (full green pods) collected from Oujda, eastern Morocco (in Mai, 2022), were dried in the shade at room temperature (25 °C) for two weeks, then powdered using an electric mixer. Powder obtained was extracted using initially boiled distilled water (infusion) with continuous steering for 1 h. After that, the suspension was filtered twice with cotton and filter paper (Watman No. 4). The filtrate was dried in a ventilated oven at 40 °C for one day and the obtained dry extract was kept away from light at 4 °C until use.

#### 2.2.2. Preparation of *O. basilicum* Hydro-Ethanolic Extract

*O. basilicum* was purchased from an herbalist in Oujda city and identified by a botanist. For preparing the hydro-ethanolic extract, the dried aerial part of the plant was reduced into fine powder using an electric blender. After that, 20 g of powder was extracted using the percolation method with ethanol/water (30/70: *v*/*v*) as the solvent. This method is more efficient than maceration and has the advantage of continually replacing the saturated solvent with a fresh one [32]. The obtained hydro-ethanolic extract was filtered and dried using the above-mentioned procedure.

### 2.3. Determination of Total Phenolics, Flavonoids and Tannins Content

The method described in our previous work [30] was used to estimate the amount of polyphenols in each extract. For that, a diluted sample (500 µL) was incubated (at room temperature, in the dark) with 250 µL of Folin–Ciocalteu reagent and saturated sodium carbonate (20%). After 30 min, the absorbance was measured using UV-Vis spectrophotometer at 725 nm. The blank contained distilled water instead of the sample. Rosmarinic acid was used to establish the calibration curve, and the results were expressed in mg rosmarinic acid equivalent/g dry extract.

The aluminum chloride method was used to determine the amount of flavonoids in *C. siliqua* and *O. basilicum* extracts. In brief, the sample (0.5 mg/mL) was incubated with aluminum chloride (0.13%) and sodium acetate (4%) reagents for 30 min, and the absorbance of the yellowish-colored product was measured at 430 nm using a UV-Vis spectrophotometer (RAY LLEIGH VIS-7220-G). The blank contained extract (0.5 mg/mL) and distilled water. A calibration curve was established using increasing concentrations of rutin, and the results were expressed in mg rutin equivalent/g dry extract.

The amount of condensed tannins available in the carob and basil extracts was determined using the method of vanillin-HCl as described previously in [33] with some modifications. Each 200 µL of sample at 10 mg/mL was incubated with 1.5 mL of vanillin (4% in methanol) and 750 µL of hydrochloric acid (37%). After agitation and 15 min incubation in a water bath at 25 °C, absorbance was measured at the 500 nm wavelength (RAY LLEIGH VIS-7220-G). For each sample, the blank contained 0.2 mL of sample and 1.5 mL of distilled water in place of the vanillin reagent. The measurements were carried out in triplicate, and the amount of condensed tannins (mg catechin equivalent/g dry extract) was determined using the calibration curve of catechin.

### 2.4. HPLC-DAD Phenolic Profile of C. siliqua and O. basilicum Extracts

#### 2.4.1. *C. siliqua* HPLC-DAD Phenolic Profile

The major polyphenol compounds present in the aqueous extract of *C. siliqua* were determined using an Agilent 1100 series HPLC chromatograph equipped with a reverse phase (C18 column, 250 × 4 mm, particle size 5 µm) and a diode array detector (DAD: Waters. 2996). The sample (30 mg) was solubilized in 3 mL of ultra-pure water (purchased from Sigma Aldrich, St. Louis, MO, USA) and filtered twice using a 0.45 µm filter (Millipore, Burlington, MA, USA). An amount of 10 µL of the sample was eluted using a solvent gradient of the mobile phase A (water + 0.5% formic acid) and B (methanol). The solvent gradient was 95% A/5% B at 0 min, 65% A/35% B at 20 min, 50% A/50% B at 25 min, 5% A/95% B at 40 min, and 95% A/5% B at 42 min. The flow rate was 1 mL/min and the temperature 20 °C. Identification of the phenolic molecules was achieved using external standards: naringenin, gallic acid, catechin, quercetin, and vanillic, syringic, and cinnamic acids (purity > 98%) eluted using the same protocol.

#### 2.4.2. *O. basilicum* HPLC-DAD Phenolic Profile

The hydro-ethanolic extract of *O. basilicum* was prepared using the same manner as for the *C. siliqua* pulp extract. However, here the sample (10 µL) was eluted using a solvent gradient constituted by mobile phases A (ultra-pure water acidified with acetic acid (pH = 3)) and B (acetonitrile): 0–1 min: 0–3% B, 1–45 min: 3–40% B, 45–55 min: 40% B, 55–56 min: 0% B. The flow rate was 1 mL/min, and the chromatogram was recorded at 320 nm. The identification of each peak was made by comparing its retention time to that of the standard (chicoric, rosmarinic, caftaric, and caffeic acids) eluted following the same procedure.

### 2.5. Preparation of the CBF

The yields of crude extracts (powder) were 25% and 20%, respectively, for basil and immature carob pods. The herbal formula was prepared by mixing carob and basil extracts in a 1:1 ratio for 15 min using a mortar and pestle.

### 2.6. Antioxidant Activity Assessment

#### 2.6.1. DPPH-Radical Scavenging Capacity

The ability of the herbal mixture (CBF) to scavenge DPPH radicals was studied using the method described in our previous work [30]. Briefly, 50 µL of the sample in increased concentrations (0.5, 1, 2, 4, 8, 16, 32, and 64 µg/mL), solubilized in distilled water, was incubated with 950 µL of DPPH reagent in methanol (0.004%), in the dark, at room temperature for 15 min. The absorbance was monitored at 517 nm against the blank (water in place of the sample). Butylated hydroxyanisole (BHA) was used as a reference antioxidant compound. The inhibition percentage was calculated using Formula (1), and curves of inhibition (%) against sample concentration were drawn. Finally, the median inhibition concentration (IC_50_) was calculated from the equation of each curve.

Inhibition (%) = (Absorbance of the blank − Absorbance of the sample/Absorbance of the blank) × 100(1)

#### 2.6.2. Ferric Reducing Antioxidant Power Assay

The capacity of the CBF to reduce the ions Fe^3+^ into Fe^2+^ was investigated as previously described in [34], with some modifications. The CBF or ascorbic acid (0.25 mL) at increasing concentrations (3.5–250 µg/mL) were mixed with the same volume of phosphate buffer (200 mM; pH = 6.6) and 0.25 mL of 1% potassium ferricyanide (K_3_Fe(CN)_6_). Tubes were incubated in a water bath at 50 °C for 20 min, and 0.25 mL of trichloroacetic acid (10%) was added, followed by 1 mL of distilled water. After the addition of 250 µL of ferric chloride (FeCl_3_, 0.1%), each tube was vigorously shaken and the absorbance was determined at 700 nm. All measurements were taken in triplicate, and water replaced the sample for the blank. Curves of absorbance against sample concentration were drawn, and from these the effective concentration (EC_50_) value (the concentration that provides the value 0.5 of absorbance) was determined using linear regression.

#### 2.6.3. Effect of the CBF on Plasma Lipoprotein Oxidation

The preventive effect of the CBF against lipoprotein-rich plasma (LPR) oxidation was conducted according to our previous report, with minor modifications [30]. Briefly, LRP was obtained from mice 24 h after administering a unique dose of an inhibitor of lipoprotein lipase, Triton WR-1339 (tyloxapol, 200 mg/kg body weight), intraperitonially. Three series of tubes were prepared: Control: 0.05 mL of LRP and 0.15 mL of distilled water. Oxidized LRP (OL): 0.05 mL of LRP incubated with 0.1 mL of copper sulfate (0.4 mg/mL) and 0.05 mL of distilled water. Test: 0.05 mL of LRP was incubated with 0.1 mL of copper sulfate reagent and 0.05 mL of the CBF or ascorbic acid (as a standard) in increasing concentrations (10, 20, 40, 80, 160, 320, and 640 μg/mL). After agitation, the tubes were incubated for 24 h (at 37 °C); 0.5 mL of thiobarbituric acid (0.8%) and 0.5 mL of trichloroacetic acid (20%) were added. The tubes were incubated in a water bath at 90 °C/30 min and cooled at room temperature for another 30 min. After a centrifugation step (10,000 rpm/5 min), the absorbance was determined at 532 nm. The concentrations of MDA were calculated via the MDA calibration curve (y = 0.0067x − 0.01, R_2_ = 0.9970). Formula (2) was used to determine the inhibition level of LRP oxidation (%):

Inhibition (%) = ([MDA] OL − [MDA] Test)/[MDA] OL × 100(2)

The IC_50_ values were determined by tracing the curves of inhibition (%) against MDA level (nM).

### 2.7. Acute Oral Toxicity Study

In order to evaluate the toxicity of the CBF, two groups of mice were used. After a one-week acclimatization period, the animals were assigned to two groups (*n* = 6): a control and a treated group (3 male and 3 female mice in each group). The protocol was in accordance with the OECD guidelines [35] for acute oral toxicity testing in rodents. Following a 12 h fasting period, the first group (*n* = 6), which served as the control, was orally administered distilled water only. Two animals from the treated group (*n* = 6) received orally a single high dose of the CBF (2000 mg/kg). If no sign of toxicity or mortality occurred after one hour, four additional animals were gavaged with the CBF at the same dose. Animals were observed with attention for the first 4 h and at 24 h and daily for 2 weeks to monitor the occurrence of any signs of toxicity such as change in behavior, body weight loss, ataxia, and apathy, as well as mortality in the animal population.

### 2.8. Study Design to Investigate the Hypolipidemic Activity

To investigate the hypolipidemic effect of the CBF, 8–10-week-old mice (23–28 g) bred in the animal house (Faculty of Sciences, Oujda, Morocco) were maintained in a controlled room (12 h light/dark cycle, 23 ± 3 °C). The animals were kept on a standard pellet diet (Society Alf Sahel, Meknes, Morocco), with free access to water and food. Animals were handled and treated according to the Directive 2010/63/EU guidelines of laboratory use [36]. After a one-week acclimatization period, the animals were divided into four groups (*n* = 6 mice/group). Normolipidemic control (NLC): mice were fed a normal show diet and gavaged daily with distilled water. Hyperlipidemic control (HLC): mice were fed the HFD ad libitum and gavaged daily with distilled water; the HFD was prepared as indicated previously [30]. CBF-treated group (CBFG): here, mice were fed the HFD ad libitum and administered the CBF by oral gavage (200 mg/kg/day). Fenofibrate-treated group (FFG): mice were fed the HFD and dosed orally with the fenofibrate drug at 100 mg/kg/day. At the end of the experiment (10 weeks), the mice were fasted for 12 h, and blood was collected from the retro-orbital sinus (slight anesthesia was administered using diethyl ether) in tubes containing an anticoagulant (trisodium citrate, 4%). After centrifugation (10,000 rpm/5min), plasma was recuperated and conserved for further analysis. The mice were sacrificed, and the liver, adipose tissue, heart, and kidneys were excised, immediately weighed, and stored at −20 °C.

#### 2.8.1. Plasma Lipid Profile Analysis

Plasma levels of TC, TG, LDL-C, HDL-C, and glucose were measured using enzymatic kits. Briefly, 10 µL of plasma was incubated with 1 mL of TC, TG, LDL-C, and HDL-C specific enzymatic kits (Biosystems Barcelona, Barcelona, Spain). The plasma level of each parameter (expressed in mg/dL) was determined from standards of known concentration by using Formula (3).

Concentration (mg/dL) = Absorbance of the sample/Absorbance of the standard × Concentration of the standard(3)

#### 2.8.2. Determination of the Atherogenic Index and LDL-C/HDL-C Ratio

As indicators of cardiovascular disease risk, the atherogenic index and LDL-C/HDL-C ratio were determined using Formulas (4) and (5), respectively:

Atherogenic index = (TC-HDL-C)/HDL-C(4)

LDL-C/HDL-C ratio = Concentration of LDL-C/Concentration of HDL-C(5)

#### 2.8.3. Liver Lipid Extraction and Analysis

To extract lipids from mouse livers, 1 g of tissue from each mouse was well homogenized in cold isopropanol (1/10, *w*/*v*) and kept at 4 °C for 24 h. Then, the obtained liver homogenates were centrifuged at 2500 rpm/5 min to obtain the supernatant. After that, 10 µL of sample and 1 mL of the TC or TG-specific enzymatic kits were incubated at 37 °C for 10 min. The absorbance measurement was taken at 500 nm. Concentrations of TC and TG were expressed in mg/g liver tissue.

### 2.9. In Silico Molecular Docking Study

The three-dimensional crystal structures of the targets involved in the regulation of lipid metabolism were downloaded from the protein data bank at https://www.rcsb.org/ (accessed on 30 July 2024) database in PDB format. This includes the structures of HMG-CoA reductase (PDB: 1HWK), PPARγ (PDB: 1I7I), PPARα (PDB: 1I7G), RXRα (PDB: 1FM6), PCSK9 (PDB: 6U26), LPL (PDB: 6E7K), LXRα (PDB: 2ACL), FAS (PDB: 1XKT), Cyp7a1 (PDB: 3DAX), C/EBPα (PDB: 8K8C), NPC1L1 (PDB: C13QNT), and ACLY (PDB: 3MWD). The SDF files of the CBF-identified phenolics were downloaded from the PubChem database at https://pubchem.ncbi.nlm.nih.gov/ (accessed on 30 July 2024) and transferred to PDB format using PyMOL (Schrodinger, version 3.0.4). AutoDockTools (version 1.5.6) software was used to prepare the receptors by removing water molecules and any co-crystalized ligands, followed by the addition of hydrogen atoms, charges, and AD4 type. Both targets and ligands were registered in PDBQT format using AutoDockTools. Molecular docking was performed using AutoDock/Vina [37], and Discovery Studio (BIOVIA, version 2024) software was used to visualize the 3D and 2D structures of the ligand–receptor complex.

### 2.10. Statistical Analysis

The software IBM SPSS statistics (version 25) was used to analyze the obtained results, and graphs were drawn using GraphPad Prism (version 8.0.2). The Shapiro–Wilk test was used to confirm the normal distribution of the data. Statistical analysis was carried out using the Student *t*-test and one-way ANOVA followed by Tukey’s post hoc test. *p* values < 0.05 were considered as statistically significant.

## 3. Results

### 3.1. Phytochemistry

#### 3.1.1. Total Polyphenols, Flavonoids, and Tannins

The total amount of polyphenols varied greatly in the extracts of the studied plants. In fact, the aqueous extract of *C. siliqua* unripe pods contained a high level of polyphenols (151 mg/g dry extract) compared to the hydro-ethanolic extract of *O. basilicum* (63 mg/g dry extract). Similarly, as indicated in Table 1, the carob extract contained a higher amount of condensed tannins (21.89 mg/g) as compared to the basil one (only 0.9 mg/g). However, both extracts had nearly the same quantity of flavonoid compounds (20 mg/g and 19 mg/g, for *C. siliqua* and *O. basilicum*, respectively).

#### 3.1.2. HPLC Phenolic Profile Analysis

**Figure 1:**
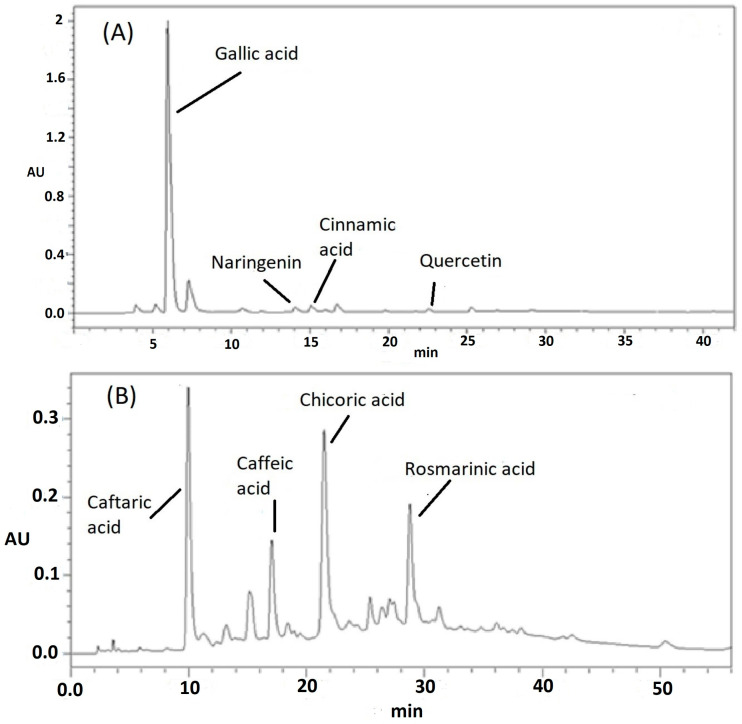
HPLC phenolic profiles of *C. siliqua* (**A**) and *O. basilicum* (**B**) extracts.

As seen in Figure 1A, the HPLC-DAD analysis of *C. siliqua* pod extract showed the presence of many phenolic compounds. Among them, gallic acid was the main constituent (74.17%). Additionally, cinnamic acid (1.31%), naringenin (0.17%), and quercetin (0.01%) were also identified using the available standards. On the other hand, the HPLC analysis of *O. basilicum* hydro-ethanolic extract (Figure 1B) revealed chicoric acid (25.94%), caftaric acid (24.45%), rosmarinic acid (13.68%), and caffeic acid (9.85%) as the main phenolic constituents.

**Figure 2:**
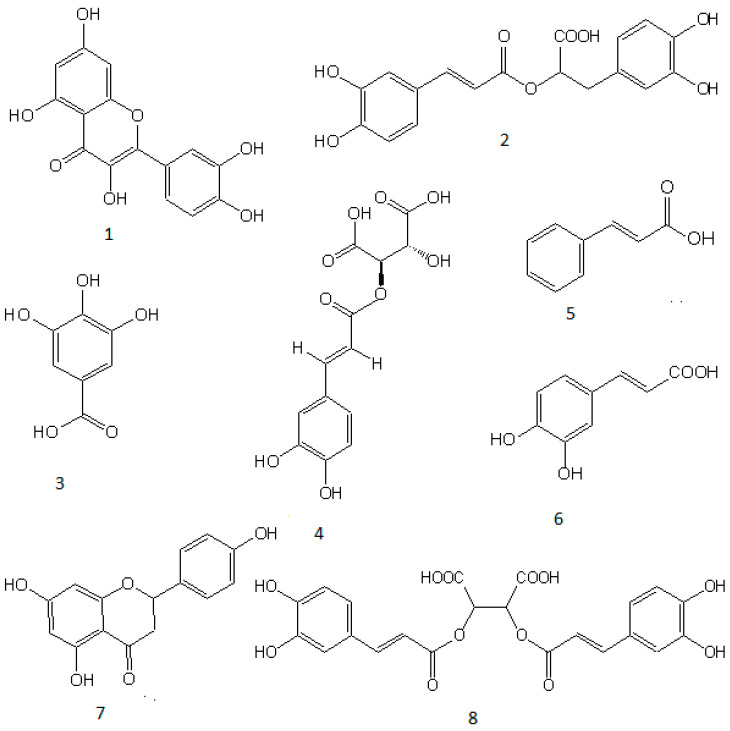
Chemical structures of the main phenolics from the immature carob pod aqueous extract and sweet basil hydro-ethanolic extract. 1: Quercetin (CID: 5280343); 2: rosmarinic acid (CID: 5281792); 3: gallic acid (CID: 370); 4: caftaric acid (CID: 64409397); 5: cinnamic acid (CID: 444539); 6: caffeic acid (CID 689043); 7: naringenin (CID: 439246); 8: chicoric acid (CID:528176) (Drawn using ChemDraw Pro 12 software).

Figure 2 presents the chemical structures of the identified phenolic molecules.

### 3.2. Antioxidant Activity

#### 3.2.1. DPPH Radical Scavenging Capacity and FRAP Assay

**Figure 3:**
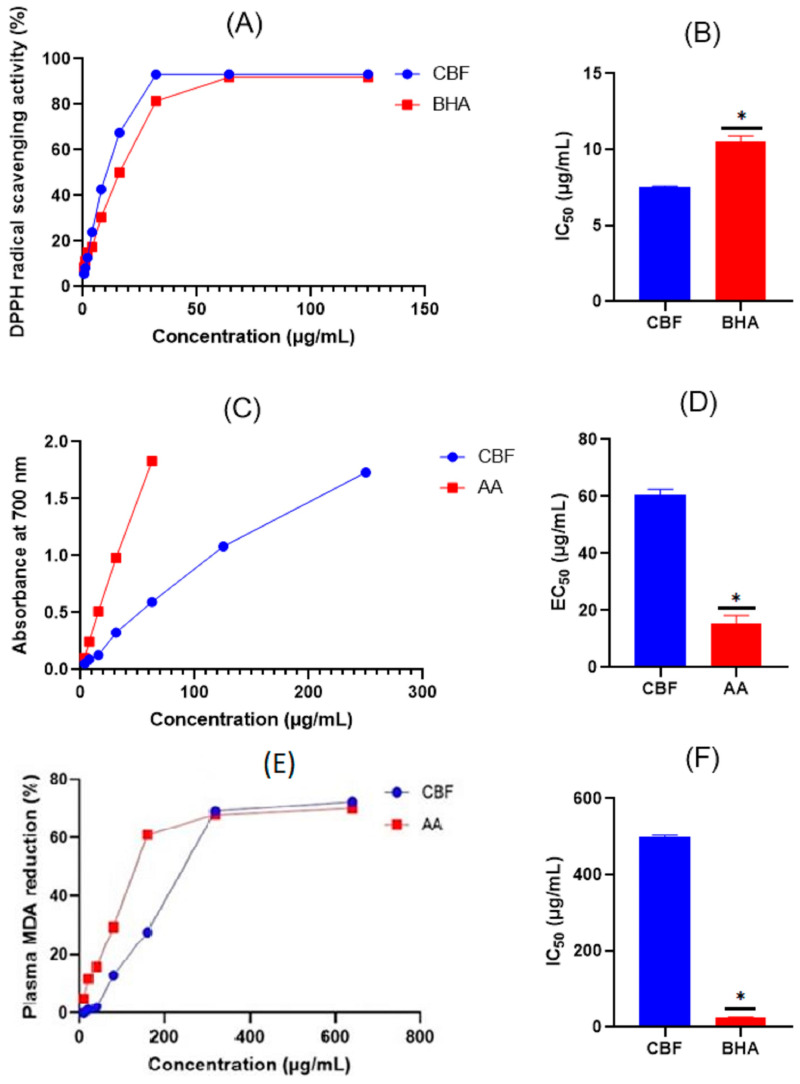
Concentration-dependent DPPH radical scavenging activity of the CBF and BHA (**A**) and the obtained IC_50_ values (**B**). Ferric reducing power activity of the CBF and AA (**C**) and the obtained EC_50_ values (**D**). Effect of CBF and AA on mice LRP oxidation (**E**) and the corresponding IC_50_ values (**F**). Results are represented as mean ± SEM (*n* = 3). CBF: carob/basil formula; AA: ascorbic acid; BHA: butylated hydroxyanisole. * *p* < 0.001.

Compared to BHA, the CBF demonstrated a dose-dependent neutralizing effect of DPPH free radicals (Figure 3A). Interestingly, as depicted in Figure 3B, the herbal formula (CBF) demonstrated better radical scavenging capacity (IC_50_ = 7.58 ± 0.01 µg/mL, *p* < 0.01) than the synthetic antioxidant BHA (IC_50_ = 10.52 ± 0.43 µg/mL).

As demonstrated in Figure 3C, the CBF displayed a high capacity to reduce ferric ions. In fact, absorbance values were 0.05, 0.09, 0.13, 0.32, 0.60, 1.08, and 1.73, respectively, at 3.5, 7, 15.5, 31, 62, 125, and 250 µg/mL. Similarly, the standard antioxidant compound AA raised the absorbance in a dose-dependent manner from 0.1 to 1.83 at concentrations ranging between 3.5 and 62 µg/mL. However, as seen in Figure 3D, the FRAP EC_50_ value of the standard AA (15 µg/mL) was significantly lower than that of the CBF (EC_50_ = 60 µg/mL).

#### 3.2.2. Effect of the CBF on LRP Oxidation

As is represented in Figure 3E, the CBF and AA prevented LRP oxidation in a concentration-dependent manner. As illustrated in Figure 3F, the CBF had an IC_50_ value of 490 ± 5 μg/mL, although this value was significantly lower than that obtained by the reference compound AA (IC_50_ = 149 ± 3 μg/mL).

### 3.3. Acute Oral Toxicity Study

In order to evaluate the safety of the CBF, we conducted acute oral toxicity testing in mice. Administering a single high dose of the CBF to mice did not cause any behavioral modifications or the apparition of any sign of toxicity as compared to the control group. Indeed, no case of mortality among animals treated orally with the CBF was recorded at the end of the 14-day study period. Thus, the median lethal dose (LD_50_), which is defined as the dose of a substance that causes death in 50% of an animal population, is estimated to be more than 2 g/kg body weight in mice. We can conclude that the CBF is relatively safe.

### 3.4. Effect of the CBF on Hyperlipidemia in Mice Fed HFD

#### 3.4.1. Body Weight and Organs Weight

The HFD significantly raised the negative control body weight gain (BWG) and adipose tissue weight (176% and 335%, respectively) compared to the NCG. Interestingly, mice orally administered the CBF exhibited a significant reduction in their BWG and adipose tissue weight after 10 weeks of daily treatment. The reduction in the BWG was 58%, and in the adipose tissue weight was 61% (Table 2). Fenofibrate exerted the same effect and lowered both the BWG (48%) and adipose tissue weight (51%) as compared to the negative control group. A significant increase in the liver weight (25%) of the negative control group was observed as compared to the standard diet-fed mice. Nevertheless, no significant difference in liver weight between the HLC and the treated groups was found. Indeed, no significant difference in heart and kidney weight was found.

#### 3.4.2. Plasma Lipid Analysis

**Figure 4:**
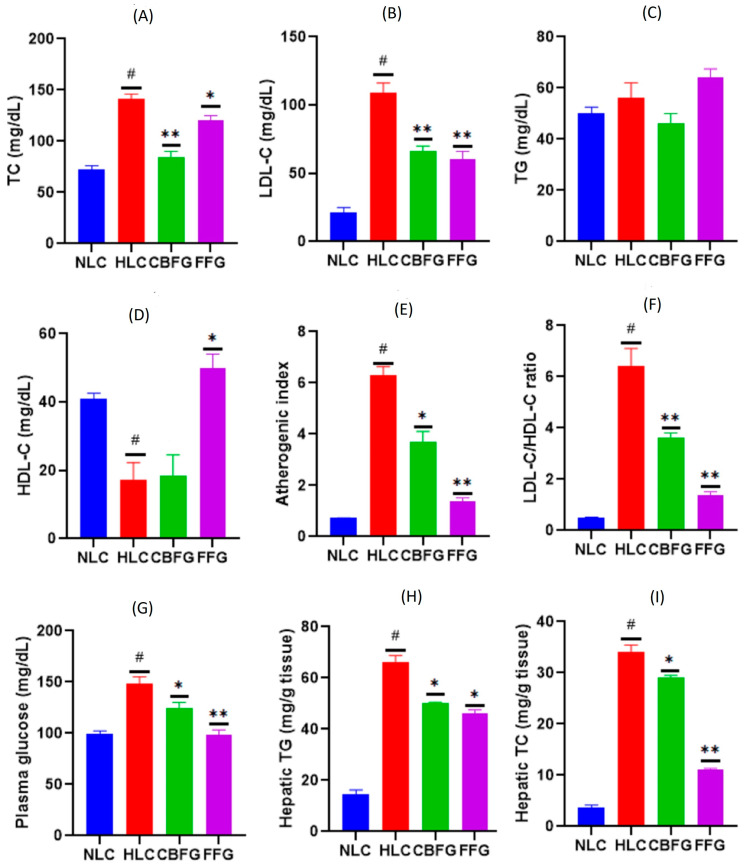
Effect of the CBF and fenofibrate on plasma TC (**A**), TG (**B**), LDL-C (**C**), and HDL-C (**D**) levels, the atherogenic index (**E**), LDL-C/HDL-C ratio (**F**), glycaemia (**G**), hepatic TG (**H**), and TC (**I**) levels in mice (*n* = 6) fed HFD for 10 consecutive weeks. NLC: normal control; HLC: hyperlipidemic control; CBFG; CBF-treated mice; FFG; fenofibrate-treated mice. ^#^ *p* < 0.001 vs. NLC; * *p* < 0.05, ** *p* < 0.01 vs. HLC.

The levels of different plasma lipid parameters (TC, TG, LDL-C, and HDL-C) obtained in the hypolipidemic study are presented in Figure 4. The HFD caused a significant increase in the negative control group plasma TC (95%) and LDL-C (419%), as well as a significant decrease in the HDL-C (58%) plasma level when compared to the normal diet-fed mice. However, animals orally administered the CBF daily for 10 weeks exhibited a significant reduction in TC (40%) and LDL-C (40%) plasma values as compared to mice fed the HFD and gavaged only with distilled water (Figure 4A,B). Fenofibrate also lowered TC (11%) and LDL-C (45%) plasma levels as compared to the HLC. Indeed, although the difference was not significant, mice fed the HFD presented 12% more plasma TG value than the NLC. The CBF decreased the TG plasma level of mice by 17% as compared to the negative control (Figure 4C). However, there was no significant difference in the HDL-C plasma level between the CBFG and the hyperlipidemic one (Figure 4D). Nevertheless, fenofibrate restored the plasma HDL-C level (66%) as compared to the HFD-fed mice.

#### 3.4.3. Atherogenic Index and LDL-C to HDL-C Ratio

HFD intake for 10 weeks resulted in a significant rise in the hyperlipidemic control group’s atherogenic index (10 folds) and LDL-C/HDL-C ratio (12 folds) as compared to mice fed a normal diet (Figure 4E,F). However, the CBF reduced dramatically the values of both the atherogenic index (53%) and the LDL-C/HDL-C ratio (41.7%) as compared to the hyperlipidemic mice. Likewise, as is presented in Figure 4E,F, mice of the fibrate-treated group showed a dramatic decrease in the values of the atherogenic index and LDL-C/HDL-C ratio as compared to the hyperlipidemic one.

#### 3.4.4. Plasma Glucose and Hepatic Lipid Analysis

The hyperlipidemic group blood glucose level increased significantly compared to the group fed the standard diet (148.23 mg/dL against 99.3 mg/dL). However, as seen in Figure 4G, mice treated orally with the CBF presented a significant decrease in their blood glucose level (16%, *p* < 0.01) compared to the HLC. Fenofibrate exerted the same effect and lowered mice glycaemia by 33% as compared to the HLC at the end of the experiment. Besides the plasma, the HFD caused a dramatic raise in the hyperlipidemic group liver TG (360%) and TC (833%) values as compared to the NLC (Figure 4H,I). However, the CBF partially alleviated triglyceride and cholesterol accumulation in the liver tissue at the end of the experiment compared to mice fed the HFD. The decrease was of about 29% and 15% for TG and TC levels, respectively, after 10 weeks’ treatment (Figure 4H,I). Likewise, fenofibrate had ameliorated considerably mice liver steatosis by the end of the experiment.

### 3.5. In Silico Molecular Docking Study of Interactions Between CBF Polyphenols and Main Proteins Involved in the Regulation of Lipid Metabolism

**Figure 5:**
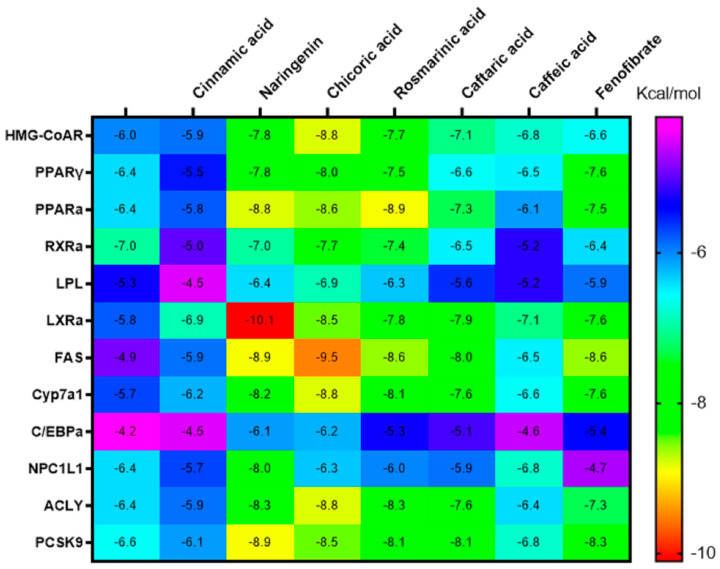
Heat map showed the binding energy (kcal/moL) between the CBF phenolics and main targets involved in the regulation of lipid metabolism. ACLY: ATP-citrate lyase; HMG−CoAR: β−hydroxy β−methylglutaryl−CoA reductase; PPAR: peroxisome proliferator activated receptors; LXRα: liver X receptor alpha; LPL: lipoprotein lipase; PCSK9: proprotein convertase subtilisin/kexin type 9; RXR: retinoid X receptors; Cyp7a1: cytochrome P450, family 7, subfamily a, polypeptide 1; C/EBPα: CCAAT/enhancer binding protein alpha; FXR: farnesoid X receptors; FAS: fatty acid synthase; NPC1L1: Niemann–Pick C1−Like 1.

In order to explore the possible mechanisms by which the CBF ameliorates lipid metabolism disorders in HFD-fed mice, we conducted a molecular docking study targeting the main proteins involved in lipid metabolism regulation. Fenofibrate was used as reference compound. The obtained results, illustrated in Figure 5, showed that this hypolipidemic drug demonstrated high affinity to FAS, PPARα/γ, PCSK9, LXRα, Cyp7a1, and ACLY (binding energy < −7 kcal/mol).

**Figure 6:**
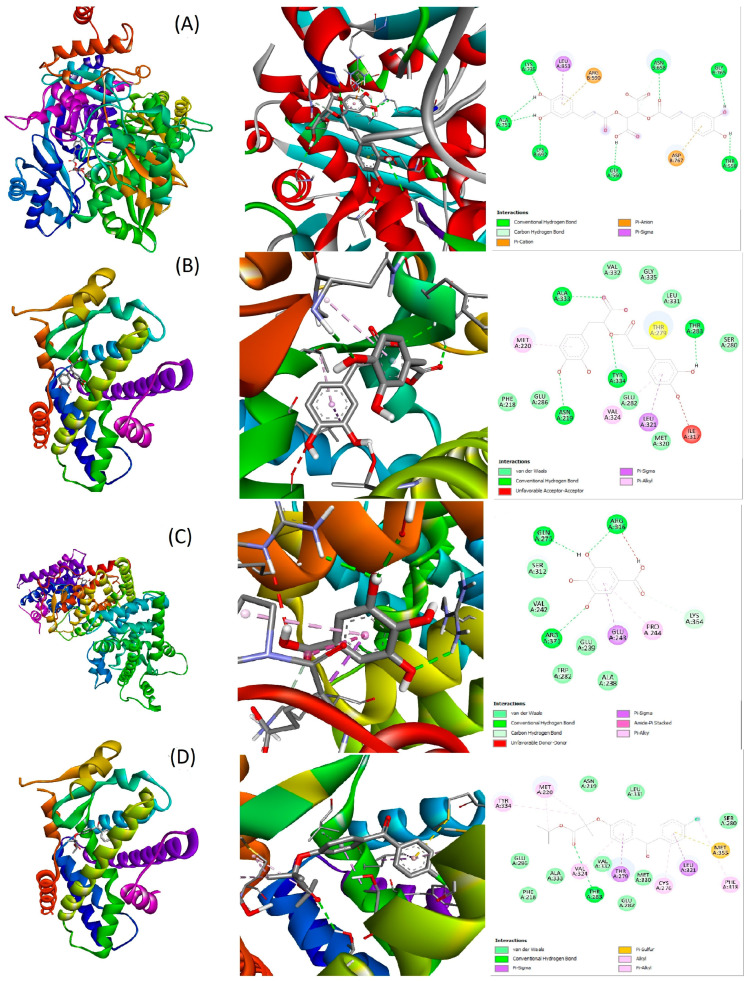
Interactions between CBF phenolics/fenofibrate and the main proteins involved in the regulation of lipid metabolism (3D and 2D structures). (**A**): chicoric acid/HMGCoA reductase; (**B**): rosmarinic/PPARα; (**C**): gallic acid/RXRα; (**D**): fenofibrate/PPARα.

**Figure 7:**
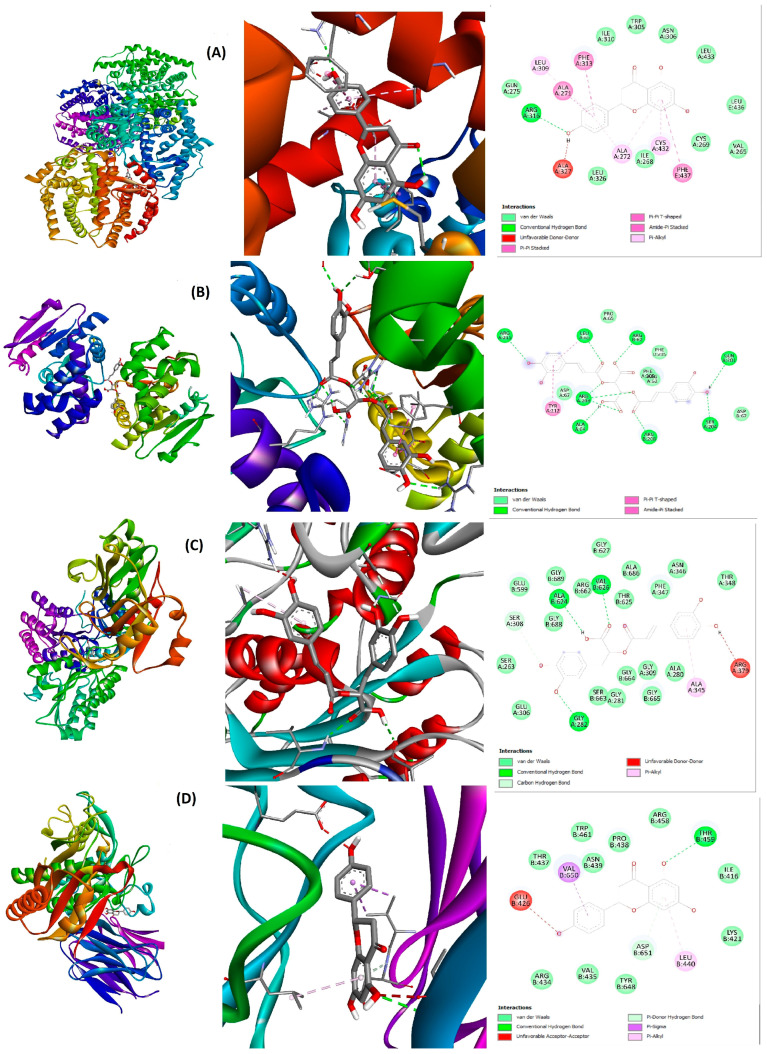
Interactions between CBF phenolics and main proteins involved in the regulation of lipid metabolism (3D and 2D structures). (**A**): naringenin/LXRα; (**B**): chicoric acid/FAS: (**C**): rosmarinic acid/ACLY; (**D**): naringenin/PCSK9.

Nevertheless, as represented in Figure 5, some CBF phenolics demonstrated a binding capacity even stronger than the reference compound fenofibrate. Interestingly, naringenin, chicoric acid, and rosmarinic acid bound strongly to HMG-CoAR, PPARα/γ, RXRα, PCSK9, LXRα, FAS, Cyp7a1, NPC1L1, and ACLY (binding scores ranged from −7 to −10.1 Kcal/mol). Indeed, caftaric acid bound to PPARα, LXRα, FAS, PCSK9, and Cyp7a1 with high affinity. These compounds could play a major role in the CBF hypolipidemic activity with a synergistic action on various pathways. As demonstrated in Figure 6 and Figure 7, the CBF phenolics as well as fenofibrate interact with the targeted proteins’ amino acid residues via conventional H-bond, carbon H-bond, pi-alkyl, pi-pi stacked, pi-sigma, pi-cation, and van der Waals interactions. However, the three other CBF phenolic constituents, gallic, caffeic, and cinnamic acids, displayed moderate affinity with the majority of ligands involved in the regulation of cholesterol and lipid metabolism (Figure 5).

## 4. Discussion

In this work, our objective was firstly analyzing the phenolic composition of *C. siliqua* unripe pods and *O. basilicum* aerial part extracts and then investigating the hypolipidemic as well as the antioxidant activity of their combination. Our findings show that the extract prepared from *C. siliqua* pods was richer in polyphenols and tannins as compared to the hydro-ethanolic extract of *O. basilicum*. Moreover, the HPLC phenolic profile revealed variability in composition between the two plants. The *C. siliqua* phenolic profile was dominated by gallic acid. Also, cinnamic acid, quercetin, and naringenin were identified using the available standards. These results were in agreement with the reports published recently by Christou and his team [19,38], in which gallic acid was the major phenolic compound available in an immature carob pulp extract. On the other hand, the hydro-ethanolic extract of *O. basilicum* aerial parts contained rosmarinic, caftaric, chicoric, and caffeic acids as major compounds. This is in accordance with many previous phytochemical studies carried out on this plant using different extraction methods [24,26,29].

The antioxidant activity of phenolic compounds extracted from various medicinal and aromatic plants is well documented. In this regard, the CBF demonstrated high capacity to neutralize free radicals in a concentration-dependent manner. This is largely attributed to the richness of carob and basil extracts in phenolic compounds. Compared to our results, Qasem and his team reported nearly the same IC_50_ value (11.23 ± 0.47 µg/mL) when investigating the DPPH radical scavenging activity of an unripe carob pulp extract [39], although other researchers found higher IC_50_ values (188.22 ± 2.23 µg/mL) [40]. Similarly, results concerning the antioxidant and lipid peroxidation inhibition of extracts prepared from basil aerial parts or from unripe carob pods have been heterogeneous [41,42]. These variations could be due not only to the method employed in preparing the extracts but also to other factors like harvest time, geographical origin, and climate conditions.

On the other hand, our findings demonstrate that the studied formulae ameliorated considerably the plasma lipid parameters, especially plasma TC and LDL-C levels in HFD-fed mice. Previous reports have demonstrated that extracts from sweet basil aerial parts ameliorated considerably blood cholesterol and LDL-C, as well as the atherogenicity index, and thereby the cardiovascular risk [25,27,28,29,43]. Also, in our recently published work, we demonstrated the considerable capacity of unripe carob pod water extract to ameliorate lipid metabolism alterations in acute and chronic models of hyperlipidemia [30].

Molecular docking has emerged as a powerful tool in drug design and discovery. This approach helps researchers to predict the possible mechanisms of action by evaluating the interaction capacity and affinity between a ligand and a receptor [31]. In the current work, we studied for the first time the in silico interactions between the main phenolic compounds of the CBF and the main proteins involved in the regulation of lipids and cholesterol metabolism. Thus, naringenin, chicoric acid, rosmarinic acid, and caftaric acid exhibited high affinity to the main enzymes involved in the cholesterol biosynthesis (HMG-CoA reductase), and absorption (NPC1L1). Moreover, these compounds bound strongly to many other proteins involved in lipid metabolism regulation, such as the LXRα, FAS, PPARα/γ, RXRα, LPL, Cyp7a1, PCSK9, and ACLY. Hence, we suggest that the CBF ameliorates the plasma lipid levels and hepatic lipid accumulation by modulating the activity of these lipid metabolism regulators. This is in agreement with a recent study that reported some potential mechanisms by which sweet basil extract could regulate lipid metabolism disorders in rats fed a high-cholesterol diet, including a reduction in the level of many proteins involved in lipid metabolism in the liver, such as FAS, LXRα, ACLY, and sterol regulatory element-binding proteins [43].

In this study, fenofibrate was used as a standard hypolipidemic agent. This drug decreases effectively the TC and LDL-C plasma levels and increases markedly the HDL-C plasma level compared to the negative control. Indeed, the molecular docking study revealed that fenofibrate bound strongly to PPARα, FAS, PPARγ, LXRα, Cyp7a1, and ACLY. According to Belakumar et al., the hypolipidemic effect of fenofibrate is mainly mediated through the activation of PPARɑ [44]. Also, this hypolipidemic agent increases the HDL-C plasma level via decreasing CETP (cholesteryl-ester transfer protein) expression [45]. On the other hand, the CBF was effective in lowering mice’s blood glucose level. The hypoglycemic activity of immature carob pods was demonstrated recently and was attributed mainly to the phenolic constituents of the tested extracts [39,40]. Additionally, a very recent work highlighted the potent capacity of unripe carob pulp extracts to inhibit α-amylase and α-glucosidase activity in vitro (IC_50_ values < 0.59 µg/mL) and decrease postprandial glycaemia in vivo [46].

Liver tissue plays a central role in cholesterol metabolism. However, accumulation of lipids in this vital organ could induce nonalcoholic fatty liver diseases (NAFLD), including steatosis and cirrhosis [5,10]. Herein, the combination between carob and sweet basil extracts reduced effectively the accumulation of lipids in the hepatic tissue after 10 weeks’ study. In a recent study, gallic acid, identified as a major phenolic compound of unripe carob pods in our study, effectively reduced the serum and hepatic lipid levels of mice fed HFD and injected with a low dose of streptozotocin as a model of NAFLD [47]. In this work, gallic acid showed moderate binding capacity to the main proteins involved in lipid metabolism. However, this phenolic acid could be metabolized into more active molecules upon entering the body, which needs more investigation. Moreover, many studies have shown that sweet basil extracts decrease effectively liver TC and TG levels [25,28,43]. The decrease in cholesterol storage in the liver could be mediated through increasing cholesterol elimination in the bile via increasing the expression of Cyp7a1, leading to bile acid biosynthesis [48]. Our in silico study revealed that rosmarinic acid, chicoric acid, and naringennin exhibited strong binding capacity not only to Cyp7a1, but also to other enzymes involved in lipid biosynthesis in the liver, including FAS and ACLY. Finally, the CBF decreased the adipose tissue weight, which could be mediated through the suppression of adipocyte differentiation via modulating C/EBPα expression [49].

## 5. Conclusions

The findings of this study demonstrate for the first time the capacity of a combination of extracts of *C. siliqua* unripe pods and *O. basilicum* aerial parts to ameliorate hypercholesterolemia and liver lipid accumulation in mice. Furthermore, the CBF exerted high antioxidant capacity and was found to be not toxic. Gallic acid, naringennin, chicoric, caftaric, and rosmarinic acids were the major phenolic phytochemicals identified through HPLC-DAD analysis. We suggest that these compounds and/or their metabolites could play a major role in the prevention of lipid metabolism disorders and related cardiovascular complications.

## Figures and Tables

**Table 1:** Extraction yield, total polyphenols, flavonoids, and tannins of *C. siliqua* and *O. basilicum* extracts.

Extract	Yield (%)	Total Polyphenols ^a^	Tannins ^b^	Flavonoids ^c^
*C. siliqua*	20%	151.5 ± 2 *	21.89 ± 0.38 *	20.14 ± 0.16 ^#^
*O. basilicum*	25%	63.4 ± 5 ^#^	0.91 ± 0.1 ^#^	19.06 ± 0.07 ^#^

Data are represented as mean ± SEM. ^a^ mg of rosmarinic acid/g dry extract; ^b^ mg of catechin/g dry extract. ^c^ mg of rutin/g dry extract. Different symbols in the same column mean the presence of a significant difference at *p* < 0.001.

**Table 2:** BWG and organ weight of mice treated orally with CBF or fenofibrate and fed HFD for 10 weeks.

Group	BWG (g)	Adipose Tissue (g)	Liver (g)	Heart (g)	Kidneys (g)
NLC	3.30 ± 0.80	0.37 ±0.04	1.6 ± 0.05	0.16 ± 0.01	0.50 ± 0.02
HLC	9.01 ± 1.99 ^#^	1.61 ± 0.2 ^##^	2.1± 0.09 ^#^	0.16 ± 0.01	0.48 ± 0.03
CBFG	3.6 ± 0.88 *	0.62 ± 0.04 **	2.04± 0.09	0.15 ± 0.01	0.45 ± 0.02
FFG	4.79 ± 1.15 *	0.83 ± 0.08 **	1.9 ± 0.3	0.15 ± 0.01	0.54 ± 0.02

NLC: normal control; HLC; hyperlipidemic control; CBFG; CBF-treated mice FFG: fenofibrate-treated mice. ^#^ *p* < 0.05, ^##^ *p* < 0.01 vs. NLC; * *p* < 0.05, ** *p* < 0.01 vs. HLC.

## Data Availability

Data are included within the article.

## Abbreviations

The following abbreviations are used in this manuscript:

AA	Ascorbic acid
ACLY	ATP-citrate lyase
BHA	Butylated hydroxyanisole
CBF	Carob/basil formula
CVD	Cardiovascular diseases
Cyp7a1	Cytochrome P450, family 7, subfamily a, polypeptide 1
DPPH	2.2-Diphenyl-1-picrylhydrazyl
EC_50_	Effective concentration
FRAP	Ferric reducing antioxidant power
HFD	High fat diet
HMG-CoAR	β-Hydroxy β-methylglutaryl-CoA reductase
HPLC	High performance liquid chromatography
IC_50_	Median inhibition concentration
LDL-C	Low density lipoprotein-cholesterol
LDH	Lactate dehydrogenase
LXRα	Liver X receptor alpha
NPC1L1	Niemann-pick-C1-Like 1
PCSK9	Proprotein convertase subtilisin/kexin type 9
PPAR	Peroxisome proliferator activated receptor
TC	Total cholesterol
TG	Triglycerides
